# Translational medicine in neuromuscular disorders: from academia to industry

**DOI:** 10.1242/dmm.041434

**Published:** 2019-10-24

**Authors:** Belinda S. Cowling, Leen Thielemans

**Affiliations:** 1 Dynacure, Pôle API, 67400 Illkirch, France; 2 2 Bridge, 2980 Zoersel, Belgium

**Keywords:** Translational research, Neuromuscular disease, Therapy, Drug development

## Abstract

Although around half of US Food and Drug Administration (FDA)-approved drugs originate from discoveries made in academic research laboratories, the US National Institutes of Health (NIH) estimates that nearly 90% of therapies developed in preclinical stages never reach clinical trials. From those in clinical trials, only 10% obtain marketing approval. Despite the recent advances in our understanding and diagnosis of neuromuscular disease, and the development of rational therapies in clinical trials, these numbers have not changed dramatically over the past two decades. This article discusses the advantages and challenges for translational research initiated from academia, and the trend towards bridging the gap between discovery and translation to the clinic. A focus is made on recent advances in therapeutic development for neuromuscular disorders.

## Abstract (precis)

**Summary:** Academia-industry partnerships are important for therapeutic development. An improved understanding of the steps required for the translation of academic discoveries will be key for future clinical success in the neuromuscular field.

## Introduction

**Fig. 1.**
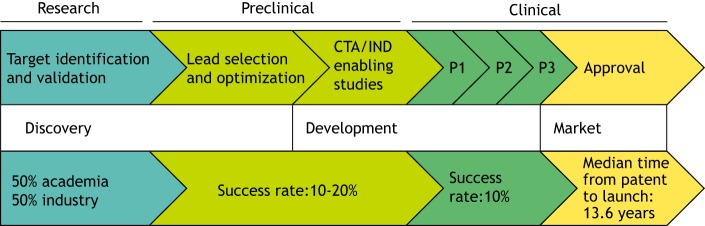
**Pathway for drug development from discovery to marketing approval.** Around 50% of new drugs were first reported by academic publications (Patridge et al., 2015). Following target identification and validation, translational scientists perform hit-finding/lead clinical candidate optimization and identification. Once the lead clinical candidate is identified, preclinical development studies are then performed to prepare for filing the appropriate applications with regulatory agencies, such as the CTA (Clinical Trial Application) in the EU or the IND (Investigational New Drug) in the USA. For neuromuscular disease, 43% of therapies in development are small molecules, whilst 23% are combined gene and antisense oligonucleotide (ASO) technologies. Nearly 50% of all neuromuscular disease therapies in development are focused on spinal muscular atrophy (SMA) and Duchenne's muscular dystrophy (DMD). If permitted, drugs may then be tested in phase 1/2/3 (P1/P2/P3) clinical trials. The median time from first patent (discovery) to launch (FDA approval) of all approved drugs was 13.6 years in 2018. Statistics from Aitken et al. (2018) and Patridge et al. (2015).

Genetic diagnosis for neuromuscular disorders has rapidly improved over the past 20 years. This is due in large part to the major advancements in our understanding of the human genome, and technological developments such as next-generation sequencing, resulting in increased detection of monogenic diseases. To date, over 500 genes have been associated with neuromuscular disorders (Bonne et al., 2017). As a result, many breakthrough advances have recently been achieved in therapy development in the neuromuscular disease field. There are currently nearly 200 products in the therapeutic pipeline for neuromuscular disorders, with around a 50:50 ratio of products at preclinical versus clinical stages of development, the majority targeting amyotrophic lateral sclerosis (ALS) and Duchenne's muscular dystrophy (DMD) (Aitken et al., 2018) (Fig. 1). Of note, between 2013 and 2018 the number of molecules in clinical trials for neuromuscular disorders increased fivefold, from around 20 to 100 (Aitken et al., 2018), and the leading products have now reached marketing approval. These recent advances suggest that the field is progressing. How did this happen, and how can we improve? This progress often but not always requires the transition from an academic research discovery ecosystem to industry-led development. This relationship supports the transition from target identification to translational development, which requires additional resources and know-how in areas such as drug manufacturing, (safety) pharmacology, pharmacokinetics, toxicology, regulatory requirements, quality management, operational support and market access.

## Translation of research discoveries to the clinic

**Fig. 2.**
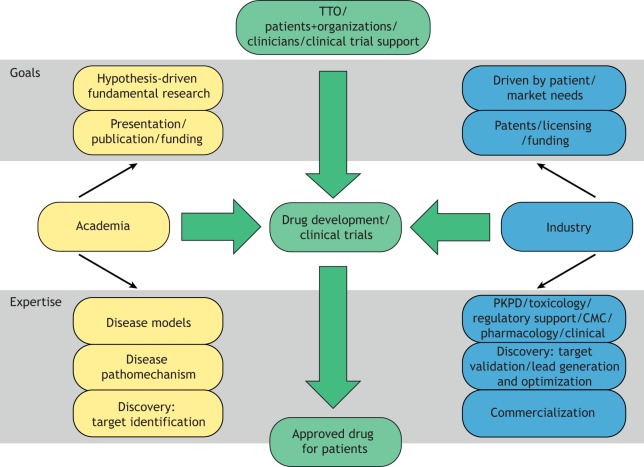
**Comparison of the goals and expertise provided by academic and industry partners.** Shown is the relationship between academia and industry. Goals and expertise for academia (yellow) and industry (blue) are highlighted. Combined expertise for drug development with input from technology transfer offices (TTOs), patient organizations and clinical trial support (green) may result in marketing approval and increased accessibility for patients. The goals and expertise listed are not mutually exclusive and may be applicable to both academia and industry. PKPD, pharmacokinetics/pharmacodynamics; CMC, chemistry, manufacturing, control.

Whilst ‘good science drives all’, there can be clear differences in motivation to perform research. Academic scientists are mainly discovery-driven, focused on formulating certain hypotheses, testing the said hypotheses, then drawing conclusions and future research perspectives. This often provides freedom for changes in research direction based on results obtained, and is limited mainly by funding, personnel and the availability of research tools. In contrast, industry-driven science is often directed by the patient needs and potential, company direction, and limitations in intellectual property (IP) and freedom to operate (Fig. 2). This process begins with defining the patient needs, e.g. the number of patients, unmet needs versus added benefit, and competitive market; this helps define the potential net value of the research program. Creating a ‘target product profile’ (TPP) during early stages of the program can be of value. A TPP is formed to summarize the relevant scientific, medical and product information. This is useful to ensure that the research and development work remains focused on the defined objectives, notably developing a safe and effective commercially available drug, and helps promote a team-based collaborative approach where all members involved in the program are aware of the common objectives. Additionally, a TPP helps determine the studies required to demonstrate efficacy and safety, both non-clinically and clinically. Industry-driven research is therefore more directed by the project needs, and if a study doesn't meet its primary endpoint, it can mean the end of the program. In an academia-industry collaboration, the limited scope of research relevant to the project needs from an industry perspective can be a source of frustration for academic scientists. Restricting research to an agreed-upon program – unless jointly agreed that changes are necessary – is, however, often required to meet the goals for industry-driven science (discussed above).

These differences in research objectives and expertise can create a gap in the knowledge chain between early discovery and translational development. Advancements have been made to try to bridge this gap. Many academic research institutions now have a technology transfer office (TTO) available to help researchers identify, evaluate and protect the potential IP of their discoveries. Following the discovery and IP protection, the TTO may then continue to support the next steps in preclinical development. This combined research and development (R&D) work involves coordination of further research studies, safety/toxicity, pharmacokinetics and pharmacodynamics (PK/PD), drug manufacturing [commonly referred to as CMC (chemistry, manufacturing, control)], regulatory input, quality, and eventually better understanding of the disease (for example, through natural history studies). Ideally, this takes place in parallel and in a well-coordinated fashion, with adequate funding and resources. If successful, this can render the program ready for the filing of an Investigational New Drug (IND) in the USA or Clinical Trial Application (CTA) in Europe. In certain academic research institutes, this is possible in-house, which has helped the advancement of many programs towards clinical readiness before licensing to biotech companies. Indeed, in 2018, around 8% of therapies in the pipeline for neuromuscular disorders were being developed by academic institutes (Aitken et al., 2018). In addition, institutions with clinicians, regulatory and non-clinical experts, and CMC expertise (on staff or accessible) have a clear additional advantage in defining the long-term vision of a project (see the TPP discussion above). However, many institutes do not have these capabilities. For researchers in such institutes, partnering with industry may be an option to provide the infrastructure and resources necessary to move a research program closer to the clinic. The TTO can facilitate the passage between academia and industry. Industry partners may, however, add limitations or constraints, such as limiting the freedom to collaborate, introducing a potential conflict of interest, and placing restrictions to IP, the freedom to disseminate the data and to request funding. This can be obstructive for academic partners for whom presenting at conferences, publishing and grants often define success. Alternatively, the pressure for academic partners to publish may hamper an open and constructive collaboration where data are shared willingly with the industry partner. Therefore, defining these points in a collaboration agreement is essential.

Recent technological advancements have helped the development of several different types of therapies. Of the nearly 200 potential therapies under development for neuromuscular diseases in 2018, around 43% were small molecules (targeting receptor modulation, epigenetic reprogramming, redox metabolism, etc.), 14% gene therapy and 9% antisense oligonucleotides (ASOs) (Aitken et al., 2018). Recent breakthroughs have been made for spinal muscular atrophy (SMA) patients, with both ASO (https://www.fda.gov/news-events/press-announcements/fda-approves-first-drug-spinal-muscular-atro phy)- and gene therapy (https://www.fda.gov/news-events/press-announcements/fda-approves-innovative-gene-therapy-treat-pediatric-patients-spinal-muscular-atrophy-rare-disease)-based treatments recently receiving US Food and Drug Administration (FDA) approval.

The availability of a particular drug is an important step in preparing for a clinical trial. This is often on the critical path (the sequence and timing of activities that predict the project end date; delay in these activities will delay the program). But what does this mean from an academic perspective? Many high-throughput drug screenings use the Prestwick chemical library (Prestwick Chemical, Inc.) or similar libraries of FDA-approved small molecules, providing initial proof of concept, such as discovery of novel potential therapeutic targets, and potentially giving new life by repurposing old drugs (Corsello et al., 2017). However, any ‘hits’ from such screens cannot always be directly tested in patients. Therefore, although drug screening is a good idea in a research setting, academic researchers should have an understanding of next steps (with regards to freedom to operate, additional preclinical/non-clinical/clinical work required) for drug development, and/or involve drug development experts at an early stage. The chemical developability and optimization potential of the ‘hit’ may also be relevant and may require collaboration with medicinal chemists to optimize the chemical formula and find new more potent variants. Any one of these factors may halt a candidate drug's development towards clinical use.

Similar issues may arise for species-specific drugs such as ASOs, where proof-of-concept work may be done against the mouse or zebrafish gene, whereas development, selection and production of a clinical candidate drug must then be performed against the human gene. This includes a battery of tests investigating preclinical safety, toxicology and pharmacological studies, which may cost millions of dollars and require access to specific technology and expertise at each step. Adeno-associated virus (AAV) vectors for gene transfer have had recent success in the clinic, prompting unprecedented interest for AAV use in drug development. However, challenges lie in producing sufficient quantities of GMP (Good Manufacturing Practices)-compliant batches of viral particles for clinical use in appropriate timelines. Several biotechnology companies (e.g. Audentes Therapeutics, Avexis, Spark Therapeutics) have invested in developing GMP in-house platforms, which may provide an advantage for R&D activities.

In parallel to ensuring development of the therapeutic compound, understanding the natural history of the disease the researchers are targeting is also important. In orphan indications, which is often the case for neuromuscular disorders, this can be essential, as little or no literature may be available. Finding the patients (in case of an orphan indication) and setting up the study can be expensive, challenging and time consuming. Working early with a network of clinicians and patient organizations can be essential in ensuring sufficient patient recruitment in a trial and selection of meaningful endpoints to measure efficacy.

## From academia to industry: bridging the gap

Many of the steps in translational development described above require not only money and resources, but also the knowledge of what/how/when to perform each step. Many undergraduate training programs focus almost entirely on what is known in a certain field (e.g. developmental biology, human genetics, etc.). However, training rarely focuses on how academic research can be translated towards the clinic. So how can young scientists learn?

Primarily, new scientists learn on the job, with each position in an industry setting providing a new set of know-how related to a specific area. Access to each division in a smaller laboratory or company can be an added advantage. Some graduate (MSc and PhD) programs allow for joint academia-industry-based positions. However, either the academic or industry partner may impose restrictions for students on the academic track who are participating in industry-sponsored work. Academic scientists must understand key steps, such as if and when to patent or publish, and develop a global vision of a project and its translational needs, which may help in project design from the initial proof-of-concept stage. Providing additional training to academic researchers highlighting these points, such as focused master's degrees on the subject, may help enhance personal development in translational science at an early stage in an individual's career.

Reproducibility, or rather potential irreproducibility, of preclinical work is important at all stages. Drug discovery may start by identifying a target and by following up with proof-of-concept work in an academic setting. A potential target may be headed for further development for clinical trials following one of the paths mentioned above. However, data generated in one lab with one model may not always be reproducible, and up to 50% of studies fail to be duplicated (Freedman et al., 2015). This has downstream effects in the drug development pipeline, which may be considerably de-risked by increasing the reproducibility of academic research. Optimizing study design, ensuring correct laboratory practices and appropriate controls, data analysis and reporting, may improve these rates. Ensuring that efficient quality systems are in place is essential. Preclinical studies performed in parallel in different laboratories/animal facilities producing similar results, and early initiation of collaboration with industry, may increase the chance of success of subsequent clinical trials. Early collaboration with industry resulting in the generation of well-documented and reproducible data may avoid the need for replication by industry partners, and consequently increase the acknowledgement to academic partners for data generated.

**Fig. 3.**
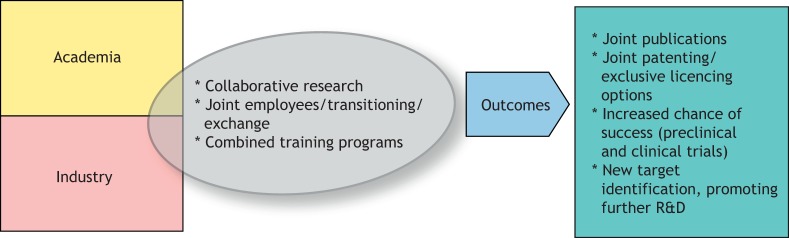
**Bridging the gap between academia and industry.** Proposed model to strengthen the relationship and improve outcomes of drug development. R&D, research and development.

How can we bridge the gap between academia and industry, and what are the real advantages of doing this? The European Medicine's Agency (EMA) has released guidelines noting the intention to strengthen the dialogue between academic research centers, drug developers and regulators at all stages of drug development by 2025 (Hines et al., 2019). Identification of a therapeutic target or mechanism of interest in the academic setting is supported by a deep understanding of the molecular mechanism of the disease and the pathway affected. This knowledge is clearly invaluable throughout the drug development process, although academic investigators should have reasonable expectations for future support and involvement, which may be limited. Initiating an early collaboration between academia and industry may increase the chances of bringing discoveries forward. Indeed, academic discoveries developed in collaboration with industry showed increased chances of success, both at the preclinical and clinical trial stages of development (Takebe et al., 2018). Furthermore, transition of personnel between academia and industry can aid the transfer of knowledge (Fig. 3). This ‘blurring of lines’ may lead to more individual researchers transitioning in the future, to the benefit of translational medicine. Improved knowledge of translational steps and the clinical picture may help academic scientists design better preclinical proof-of-concept studies, thus increasing the chances of success in the clinic.

## Recent examples of success

Around 50% of new drugs were first reported by academic publications (Patridge et al., 2015), highlighting the importance of academic research in drug discovery. Below are examples of gene therapies and ASO-mediated therapies originating from academia-industry collaborations that are now under clinical development or with recent marketing approval.

A well-studied disease in the neuromuscular field is DMD (OMIM 310200), which affects approximately 1 in 5000 male births (Mendell et al., 2012), and results in severe progressive muscle wasting and early death. Louis Kunkel's team discovered the genetic cause of the disease in 1986 (Monaco et al., 1986; Hoffman et al., 1987). The first targeted therapy for DMD, aimed at tackling expression of a functional dystrophin protein, was approved 30 years later in 2016 by the FDA. It was developed by Sarepta Therapeutics and named Exondys 51 (eteplirsen). Exondys 51 is a phosphorodiamidate morpholino oligomer (PMO) ASO that binds specifically to exon 51 of the human dystrophin pre-mRNA, resulting in restoration of the correct reading frame and thus promoting production of a truncated dystrophin protein (exon 51 skipping). This is applicable to around 13% of the DMD patient population (Aartsma-Rus et al., 2009), and approval was based on minor increases in dystrophin levels in muscle biopsies of treated patients. Confirmative studies are, however, required. Several additional approaches are now in preclinical or clinical testing stages, including exon-skipping, gene-therapy and CRISPR/Cas9-mediated approaches, suggesting that additional options may soon be available to patients. Whilst full-length dystrophin largely exceeds viral-vector packaging capabilities, AAV-mediated gene delivery of a mini or microdystrophin gene has been shown to have therapeutic potential preclinically (Harper et al., 2002; Duan, 2018), and several companies are now conducting clinical trials, including Sarepta Therapeutics (https://clinicaltrials.gov/ct2/show/NCT03375164), Solid Biosciences (https://clinicaltrials.gov/ct2/show/NCT03368742) and Pfizer (https://clinicaltrials.gov/ct2/show/NCT03362502). An alternative option is AAV-delivered CRISPR/Cas9-mediated gene editing in DMD, which was first performed in mice (Amoasii et al., 2017; Min et al., 2019) and in dogs (Amoasii et al., 2018). As a result, Exonics Therapeutics was founded by lead scientist Eric Olson to advance the DMD program and to investigate CRISPR/Cas9-mediated therapies for additional neuromuscular disorders (http://exonicstx.com/).

SMAs are rare hereditary neurodegenerative disorders resulting in progressive loss of motor neurons and muscle wasting, affecting approximately 1 in 10,000 live births (Sugarman et al., 2012). Causative recessive mutations in the *SMN1* gene, resulting in loss of SMN protein expression and SMA with varied onset and severity (SMA1 253300; SMA2 OMIM 253550; SMA3 253400; SMA4 OMIM271150), were identified in 1995 (Lefebvre et al., 1995). *SMN2* is a paralogous gene to *SMN1* that predominantly produces SMN protein lacking exon 7, resulting in rapid degradation (Lorson et al., 1999; Monani et al., 1999). ASO-mediated inclusion of exon 7 in the *SMN2* gene results in increased SMN protein expression and rescued disease features in humanized SMA mice (Hua et al., 2010). This approach was developed for the clinic by Biogen and Ionis Pharmaceuticals, and named Sprinraza (nusinersen). The drug received FDA approval in December 2016 to treat SMA patients. In parallel, an AAV-mediated SMN gene-replacement-therapy approach produced positive data in mouse models (Foust et al., 2010), prompting the development of a second therapeutic approach towards clinical trials. This approach, named Zolgensma, was developed by AveXis in collaboration with the academic team responsible for the discovery, and received provisional FDA approval for a subset of SMA patients in May 2019. The approval of both approaches, with additional therapies for SMA under development, suggests that the potential of combination therapies, and of collaboration between academia and industry, are relevant to address in neuromuscular disease research.

Congenital myopathies are rare muscle disorders affecting around 1 in 25,000 births (Amburgey et al., 2011). They are often severe with neonatal or childhood onset. A group of congenital myopathies known as centronuclear myopathies are caused by monogenic mutations in one of several genes, resulting in a myopathic phenotype of varying severity and a common histopathological appearance of abnormally localized central nuclei in muscle biopsies. The most severe X-linked form (XL-CNM; also known as myotubular myopathy; OMIM 310400) is caused by mutations in *MTM1*, encoding myotubularin (Laporte et al., 1996). Following the discovery of the genetic cause of XL-CNM in 1996, the first proof-of-concept gene-therapy approach to replace the missing gene via AAV administration was performed in mice (Buj-Bello et al., 2008) and in dogs (Childers et al., 2014). Audentes Therapeutics has now developed this program for translation, resulting in a clinical trial that started in 2017. Positive interim data have been released from this ongoing trial following a single administration of AT001 (AAV-MTM1) (http://investors.audentestx.com/news-releases/news-release-details/audentes-therapeutics-presents-new-positive-data-aspiro-clinical; https://clinicaltrials.gov/ct2/show/NCT03199469), potentially prompting an accelerated request for marketing approval.

An additional approach for CNM is under development that uses a constrained ethyl (cET) ASO to target the *DNM2* gene in patients. Following the initial discovery of this *Dnm2*-reducing approach in an academic setting (Cowling et al., 2014), a collaboration was established between the academic group, the local TTO SATT (Societies for Acceleration and Transfer of Technology, a French private TTO), Conectus, and Ionis Pharmaceuticals to develop a mouse surrogate targeting *Dnm2* and establish proof-of-concept testing (Tasfaout et al., 2017; Buono et al., 2018). Following positive data, the company Dynacure was created to develop an ASO against human *DNM2* for the treatment of CNM patients. This partnership between academia and industry should result in the initiation of a clinical trial in CNM patients in 2019 (https://www.dynacure.com/news/dynacure-announces-approval-of-clinical-trial-application-for-dyn101-an-antisense-medicine-to-treat-rare-disease-centronuclear-myopathies/; https://clinicaltrials.gov/ct2/show/NCT04033159).

These examples of recent translation from discovery of proof of concept to development towards clinical trials highlight the possibility and virtue for collaboration between various actors of the academic and industrial ecosystems. Such collaboration benefits from the complementary expertise and resources provided by each partner and can result in rapid progression from bench to bedside for patients (Fig. 3).

## Suggestions for young scientists

Specialized training at the undergraduate level is already restrictive, with early selection towards basic science. As an academic scientist, whether work is focused on basic science or translational proof-of-concept research, it is important to have a level of understanding of the potential clinical implications of the work. This can help not only with obtaining funding (by, for example, identifying the relevance to the healthcare industry), but also with the design and development of the project. The early input from a clinician working with patients may help understand the unmet medical needs (e.g. diagnosis, biomarkers, treatment). Understanding the technological capabilities linked to pharmacokinetics, a drug's formulation and its safety profile may be considered when designing a study protocol at an early stage, and may increase the long-term chances of success of the project. Based on this holistic vision, proof-of-concept studies are best designed with clinical translation in mind. Furthermore, the path from discovery to translational research does not run only in one direction, but is often bidirectional, where results obtained in developmental or clinical research may suggest or necessitate further or novel basic research, thus supporting symbiosis between the fields. Providing specialized training to young researchers in this domain will hopefully prepare scientists for drug development in an academic or industry setting.

The term ‘publish or perish’ highlights the importance placed on publishing as an acknowledgement of scientific success. Academic scientists are therefore driven to present at conferences and publish novel data as soon as possible. But what if presentation at a conference means the work is in the public domain before having been protected, thus invalidating any further patent applications? Whilst the researcher is driven to highlight the success of their work, this is contraindicative to the protection of IP and, to a lesser extent, to the discovery and further development of the work. Both are feasible and even desirable but, for research with translation potential, their timing needs to be carefully considered. Placing more importance on valorization (such as patenting) of the research, which is now being considered in more academic grant applications, should help with this step. Institutions promoting academia-industry collaborations may help by adequately recognizing and rewarding academic investigators for successful industry collaborations. Furthermore, whilst publishing may encourage individual recognition through ranking of first and senior authors or acknowledging a single presenter, teamwork is crucial in drug development due to the complexity of combining the several different areas of expertise required for successful translation. An academic environment that cultivates teamwork is an advantage for this process.

So, how can a young scientist gain an understanding of translational research? I recommend attending training opportunities on drug development and seeking out colleagues in different fields for discussion. Search for positions or projects that bridge between academia and industry, and gain experience in an academic setting that has a strong collaboration with clinicians working with patients. Many people I have interacted with have had different and often unconventional paths to industry. I personally spent over 10 years in academia before joining a biotech company in recent years. One of the surprising discoveries from my experience was gaining an appreciation that drug development is a team effort where results are shared to enable progress, and collaboration is a critical part of this process. The deep understanding that academic scientists have on disease pathogenesis and molecular mechanisms in their field of interest is invaluable throughout the drug development process. Academic scientists should evaluate their field of expertise to identify areas that are underpopulated and where there is a likely to be a future need. With the recent progress in drug development in the neuromuscular field, there is a high demand for scientists that are well trained to facilitate the advancement of these discoveries to towards the clinic.

## References

[DMM041434C1] Aartsma-Rus A., Fokkema I., Verschuuren J., Ginjaar I., van Deutekom J., van Ommen G. J. and den Dunnen J. T. (2009). Theoretic applicability of antisense-mediated exon skipping for Duchenne muscular dystrophy mutations. *Hum. Mutat.* 30, 293-299. 10.1002/humu.20918 19156838

[DMM041434C2] Aitken M., Mercer E. J. and McKemey A. (2018). Understanding neuromuscular disease care: current state and future prospects. *IQVIA Institute for Human Data Science*. https://www.iqvia.com/insights/the-iqvia-institute/reports/understanding-neuromuscular-disease-care.

[DMM041434C3] Amburgey K., McNamara N., Bennett L. R., McCormick M. E., Acsadi G. and Dowling J. J. (2011). Prevalence of congenital myopathies in a representative pediatric united states population. *Ann. Neurol.* 70, 662-665. 10.1002/ana.22510 22028225

[DMM041434C4] Amoasii L., Long C., Li H., Mireault A. A., Shelton J. M., Sanchez-Ortiz E., McAnally J. R., Bhattacharyya S., Schmidt F., Grimm D. et al. (2017). Single-cut genome editing restores dystrophin expression in a new mouse model of muscular dystrophy. *Sci. Transl. Med.* 9, eaan8081 10.1126/scitranslmed.aan8081 29187645 PMC5749406

[DMM041434C5] Amoasii L., Hildyard J. C. W., Li H., Sanchez-Ortiz E., Mireault A., Caballero D., Harron R., Stathopoulou T. R., Massey C., Shelton J. M. et al. (2018). Gene editing restores dystrophin expression in a canine model of Duchenne muscular dystrophy. *Science* 362, 86-91. 10.1126/science.aau1549 30166439 PMC6205228

[DMM041434C6] Bonne G., Rivier F. and Hamroun D. (2017). The 2018 version of the gene table of monogenic neuromuscular disorders (nuclear genome). *Neuromuscul. Disord.* 27, 1152-1183. 10.1016/j.nmd.2017.10.005 29961566

[DMM041434C7] Buj-Bello A., Fougerousse F., Schwab Y., Messaddeq N., Spehner D., Pierson C. R., Durand M., Kretz C., Danos O., Douar A. M. et al. (2008). AAV-mediated intramuscular delivery of myotubularin corrects the myotubular myopathy phenotype in targeted murine muscle and suggests a function in plasma membrane homeostasis. *Hum. Mol. Genet.* 17, 2132-2143. 10.1093/hmg/ddn112 18434328 PMC2441725

[DMM041434C8] Buono S., Ross J. A., Tasfaout H., Levy Y., Kretz C., Tayefeh L., Matson J., Guo S., Kessler P., Monia B. P. et al. (2018). Reducing dynamin 2 (DNM2) rescues DNM2-related dominant centronuclear myopathy. *Proc. Natl. Acad. Sci. USA* 115, 11066-11071. 10.1073/pnas.1808170115 30291191 PMC6205463

[DMM041434C9] Childers M. K., Joubert R., Poulard K., Moal C., Grange R. W., Doering J. A., Lawlor M. W., Rider B. E., Jamet T., Daniele N. et al. (2014). Gene therapy prolongs survival and restores function in murine and canine models of myotubular myopathy. *Sci. Transl. Med.* 6, 220ra10 10.1126/scitranslmed.3007523 PMC4105197 24452262

[DMM041434C10] Corsello S. M., Bittker J. A., Liu Z., Gould J., McCarren P., Hirschman J. E., Johnston S. E., Vrcic A., Wong B., Khan M. et al. (2017). The drug repurposing hub: a next-generation drug library and information resource. *Nat. Med.* 23, 405-408. 10.1038/nm.4306 28388612 PMC5568558

[DMM041434C11] Cowling B. S., Chevremont T., Prokic I., Kretz C., Ferry A., Coirault C., Koutsopoulos O., Laugel V., Romero N. B. and Laporte J. (2014). Reducing dynamin 2 expression rescues X-linked centronuclear myopathy. *J. Clin. Invest.* 124, 1350-1363. 10.1172/JCI71206 24569376 PMC3938268

[DMM041434C29] Duan D. (2018). Systemic AAV micro-dystrophin gene therapy for Duchenne muscular dystrophy. *Mol Ther.* 26, 2337-2356. 10.1016/j.ymthe.2018.07.011 30093306 PMC6171037

[DMM041434C12] Foust K. D., Wang X., McGovern V. L., Braun L., Bevan A. K., Haidet A. M., Le T. T., Morales P. R., Rich M. M., Burghes A. H. et al. (2010). Rescue of the spinal muscular atrophy phenotype in a mouse model by early postnatal delivery of SMN. *Nat. Biotechnol.* 28, 271-274. 10.1038/nbt.1610 20190738 PMC2889698

[DMM041434C13] Freedman L. P., Cockburn I. M. and Simcoe T. S. (2015). The economics of reproducibility in preclinical research. *PLoS Biol.* 13, e1002165 10.1371/journal.pbio.1002165 26057340 PMC4461318

[DMM041434C14] Harper S. Q., Hauser M. A., DelloRusso C., Duan D., Crawford R. W., Phelps S. F., Harper H. A., Robinson A. S., Engelhardt J. F., Brooks S. V. et al. (2002). Modular flexibility of dystrophin: implications for gene therapy of Duchenne muscular dystrophy. *Nat. Med.* 8, 253-261. 10.1038/nm0302-253 11875496

[DMM041434C15] Hines P. A., Guy R. H., Humphreys A. J. and Papaluca-Amati M. (2019). The european medicines agency's goals for regulatory science to 2025. *Nat. Rev. Drug Discov.* 18, 403-404. 10.1038/d41573-019-00071-2 31160761

[DMM041434C16] Hoffman E. P., Brown R. H. Jr. and Kunkel L. M. (1987). Dystrophin: the protein product of the Duchenne muscular dystrophy locus. *Cell* 51, 919-928. 10.1016/0092-8674(87)90579-4 3319190

[DMM041434C17] Hua Y., Sahashi K., Hung G., Rigo F., Passini M. A., Bennett C. F. and Krainer A. R. (2010). Antisense correction of SMN2 splicing in the CNS rescues necrosis in a type III SMA mouse model. *Genes Dev.* 24, 1634-1644. 10.1101/gad.1941310 20624852 PMC2912561

[DMM041434C18] Laporte J., Hu L. J., Kretz C., Mandel J. L., Kioschis P., Coy J. F., Klauck S. M., Poustka A. and Dahl N. (1996). A gene mutated in X-linked myotubular myopathy defines a new putative tyrosine phosphatase family conserved in yeast. *Nat. Genet.* 13, 175-182. 10.1038/ng0696-175 8640223

[DMM041434C19] Lefebvre S., Burglen L., Reboullet S., Clermont O., Burlet P., Viollet L., Benichou B., Cruaud C., Millasseau P., Zeviani M. et al. (1995). Identification and characterization of a spinal muscular atrophy-determining gene. *Cell* 80, 155-165. 10.1016/0092-8674(95)90460-3 7813012

[DMM041434C20] Lorson C. L., Hahnen E., Androphy E. J. and Wirth B. (1999). A single nucleotide in the SMN gene regulates splicing and is responsible for spinal muscular atrophy. *Proc. Natl. Acad. Sci. USA* 96, 6307-6311. 10.1073/pnas.96.11.6307 10339583 PMC26877

[DMM041434C21] Mendell J. R., Shilling C., Leslie N. D., Flanigan K. M., al-Dahhak R., Gastier-Foster J., Kneile K., Dunn D. M., Duval B., Aoyagi A. et al. (2012). Evidence-based path to newborn screening for Duchenne muscular dystrophy. *Ann. Neurol.* 71, 304-313. 10.1002/ana.23528 22451200

[DMM041434C22] Min Y. L., Li H., Rodriguez-Caycedo C., Mireault A. A., Huang J., Shelton J. M., McAnally J. R., Amoasii L., Mammen P. P. A., Bassel-Duby R. et al. (2019). CRISPR-Cas9 corrects Duchenne muscular dystrophy exon 44 deletion mutations in mice and human cells. *Sci. Adv.* 5, eaav4324 10.1126/sciadv.aav4324 30854433 PMC6402849

[DMM041434C23] Monaco A. P., Neve R. L., Colletti-Feener C., Bertelson C. J., Kurnit D. M. and Kunkel L. M. (1986). Isolation of candidate cDNAs for portions of the Duchenne muscular dystrophy gene. *Nature* 323, 646-650. 10.1038/323646a0 3773991

[DMM041434C24] Monani U. R., Lorson C. L., Parsons D. W., Prior T. W., Androphy E. J., Burghes A. H. M. and McPherson J. D. (1999). A single nucleotide difference that alters splicing patterns distinguishes the SMA gene SMN1 from the copy gene SMN2. *Hum. Mol. Genet.* 8, 1177-1183. 10.1093/hmg/8.7.1177 10369862

[DMM041434C25] Patridge E. V., Gareiss P. C., Kinch M. S. and Hoyer D. W. (2015). An analysis of original research contributions toward FDA-approved drugs. *Drug Discov. Today* 20, 1182-1187. 10.1016/j.drudis.2015.06.006 26113307

[DMM041434C26] Sugarman E. A., Nagan N., Zhu H., Akmaev V. R., Zhou Z., Rohlfs E. M., Flynn K., Hendrickson B. C., Scholl T., Sirko-Osadsa D. A. et al. (2012). Pan-ethnic carrier screening and prenatal diagnosis for spinal muscular atrophy: clinical laboratory analysis of >72,400 specimens. *Eur. J. Hum. Genet.* 20, 27-32. 10.1038/ejhg.2011.134 21811307 PMC3234503

[DMM041434C27] Takebe T., Imai R. and Ono S. (2018). The current status of drug discovery and development as originated in united states academia: the influence of industrial and academic collaboration on drug discovery and development. *Clin. Transl. Sci.* 11, 597-606. 10.1111/cts.12577 29940695 PMC6226120

[DMM041434C28] Tasfaout H., Buono S., Guo S., Kretz C., Messaddeq N., Booten S., Greenlee S., Monia B. P., Cowling B. S. and Laporte J. (2017). Antisense oligonucleotide-mediated Dnm2 knockdown prevents and reverts myotubular myopathy in mice. *Nat. Commun.* 8, 15661 10.1038/ncomms15661 28589938 PMC5467247

